# Efficacy and safety of So-Cheong-Ryong-Tang in patients with atopic dermatitis and respiratory disorders: Study protocol of a double-blind randomized placebo-controlled trial

**DOI:** 10.1097/MD.0000000000018565

**Published:** 2020-01-10

**Authors:** Su-Jin Kang, Han-Baek Cho, Eun-Heui Jo, Geum-Jin Yang, Ji-Eun Hong, Ju-Hyun Lee, Yu-Hwa Shim, Jung-Hyun Mun, Min-Cheol Park

**Affiliations:** a Department of Korean Medicine Obstetrics & Gynecology, Won-Kwang University Korean Medicine Hospital, Iksan-si; b Department of Acupuncture and Moxibustion, Won-Kwang University Korean Medicine Hospital, Deokjin-gu, Jeonju-si; c Korean Medicine Dermatology Clinical Research Center, Won-Kwang University; d Department of Korean Medicine Ophthalmology and Otolaryngology and Dermatology, Won-Kwang University Korean Medicine Hospital; e Won-Kwang University Korean Medicine Hospital, Iksan-si, Jeollabuk-do, Republic of Korea.

**Keywords:** atopic dermatitis, atopic eczema, herbal medicine, randomized controlled trial, Sho-Seiryu-To, Socheongryong-Tang, So-Cheong-Ryong-Tang, Xiao-Qing-Long-Tang

## Abstract

**Background:**

Atopic dermatitis (AD, atopic eczema) is a pruritic, inflammatory, chronic skin disease. Since there is limitation of conventional treatment of AD, traditional herbal medicine can be an attractive therapeutic option in patients having AD for a long time. So-Cheong-Ryong-Tang (SCRT) has been found to inhibit histamine release and degranulation of mast cells, differentiation of basophils, and proliferation of eosinophils. We designed this clinical trial to evaluate the efficacy and safety of SCRT as compared to placebo in patients with AD and respiratory disorders.

**Methods/design:**

This study is a single-center, randomized, double-blind, placebo-controlled, and investigator-initiated clinical trial. A total of 60 patients between 7 and 65 years of age with AD and respiratory disorders who received a diagnosis of AD by Hanifin and Rajka criteria who scored 15 to 50 in a scoring atopic dermatitis (SCORAD) will be enrolled. Participants will be randomly assigned to the SCRT or placebo group in a ratio of 1:1 and they will have a visit schedule comprising 4 visits including a screening visit during 8 to 10 weeks. The participants will be administered SCRT or placebo 3 times a day for 4 weeks. The primary outcome will be measured by a change of the SCORAD index. The secondary outcomes will be measured by changes in the dose and frequency of usage of the AD ointment, dermatology life quality index scores, pruritus and sleep disorder in visual analog scale, skin moisture content, skin surface temperature, Hamilton anxiety rating scale scores, depression rating scale scores, stress/autonomic nervous function test, and attention deficit hyperactivity disorder survey scores at week 4 as compared to those at the baseline.

**Discussion:**

To the best of our knowledge, SCRT has rarely been reported for dermatologic diseases. This will be the first clinical trial to assess the efficacy and safety of SCRT in patients with AD and respiratory disorders. We hope that the results of this trial will provide evidence for the use of SCRT as a new treatment for AD with respiratory disorders.

**Trial registration:**

Korean National Clinical Trial Registry, Clinical Research Information Service. (KCT0004148) (https://cris.nih.go.kr/cris/search/search_result_st01_en.jsp?seq=14981&ltype=&rtype=).

## 1 Introduction

Atopic dermatitis (AD, atopic eczema) is a pruritic, inflammatory, chronic skin disease affecting up to 20% of children and 2% to 8% of adults in a majority of the countries worldwide.^[1]^ Although the prevalence of AD varies across countries, it is increasing both in the industrialized and low-income countries.^[2]^ Family history of atopic disorders including rhinitis and asthma is an important risk factor for the development of AD.^[3]^ Moreover, AD in infancy can progress to the development of asthma and allergic rhinitis, and this sequence is called the atopic march.^[4]^ The pathogenesis of AD is not completely understood; however, it is generally accepted that dysfunction of the skin barrier and hyper-immune activation are related to the onset of the disease.^[5]^ In the acute phase of AD, increased IgE production and eosinophilia are observed and are associated with increased expression of TH2 cytokines, interleukin (IL)-4, IL-5, and IL-13.^[6]^

The optimal treatment of AD consists of avoiding triggering factors, maintenance of skin hydration, restoration of the skin barrier function, patient education, and pharmacologic options, such as topical corticosteroids, and topical calcineurin inhibitors.^[7]^ However, long term use of systemic corticosteroids can result in severe adverse effects, such as hypothalamic-pituitary-adrenal axis suppression, growth suppression in children, osteoporosis, glucose intolerance, Cushing syndrome, opportunistic infections, and rebound flaring.^[8]^ Further, some patients remain unresponsive to conventional treatment.

Traditional herbal medicine (THM) has been widely used in East Asian countries for thousands of years to treat various diseases.^[9]^ For the treatment of AD, 74% of patients in Korea have used THM as an alternative therapy.^[10]^ THM can be an attractive therapeutic option in patients having AD for a long time. So-Cheong-Ryong-Tang (SCRT, Xiao-Qing-Long-Tang in China or Sho-Seiryu-To in Japan) is the mixture of the following 8 herbs: *Epoedrae Herba*, *Paeoniae Radix* Alba, *Schizandrae Fructus*, *Pinelliae Rhizoma*, *Asari Herba Cum Radice*, *Zingiberis Rhizoma*, *Cinnamomi Ramulus*, and *Glycyrrhizae Radix*.^[11]^ SCRT has been used clinically for the treatment of various respiratory diseases, such as allergic rhinitis, bronchial asthma, and common cold.^[12]^

SCRT has been found to inhibit histamine release and degranulation of mast cells,^[13]^ differentiation of basophils,^[14]^ and proliferation of eosinophils.^[15]^ Furthermore, it is reported that SCRT regulates Th1/Th2 immune activity under Th2 dominant condition.^[16]^ To the best of our knowledge, SCRT has rarely been reported for dermatologic diseases, except for a case report and a clinical trial that studied the efficacy of SCRT in conjunction with electrical stimulation in patients with AD.^[17,18]^ However, no studies have evaluated the effects of SCRT monotherapy in patients with AD and respiratory disorders. Hence, we designed this trial to evaluate the efficacy and safety of SCRT as compared to placebo in patients with AD and respiratory disorders.

## 2 Methods

### 2.1 Study design

**Figure 1:**
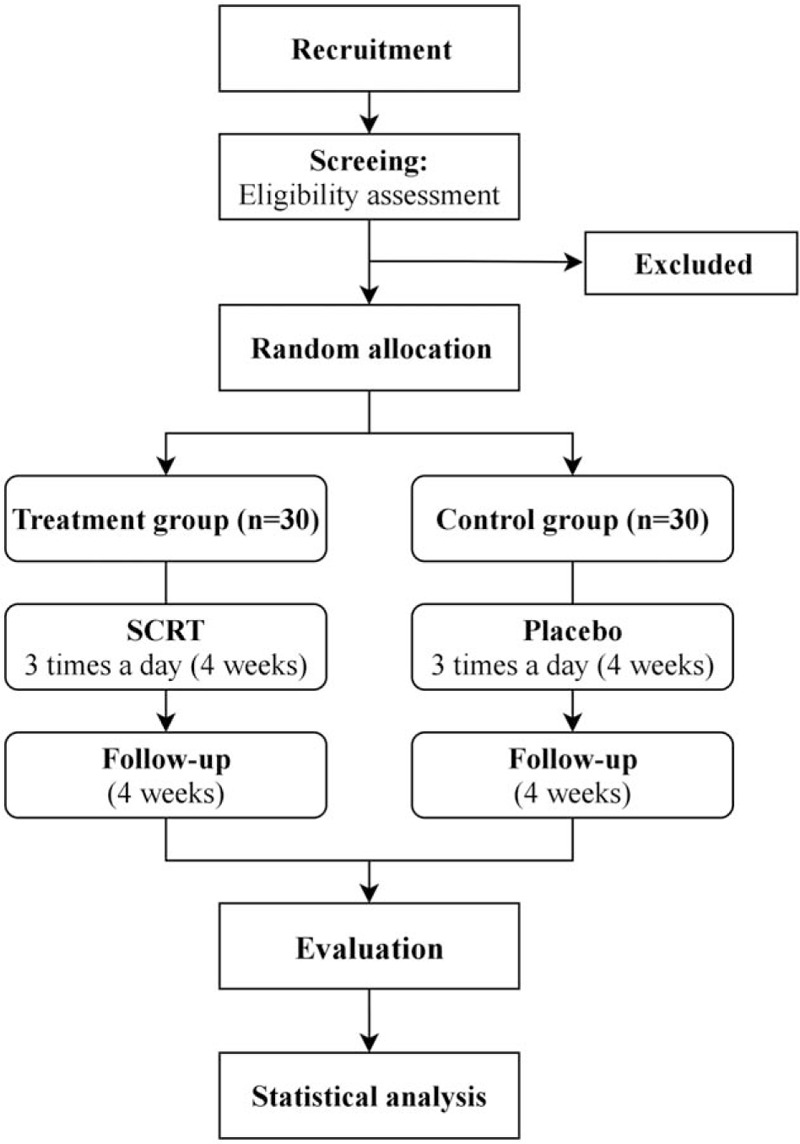
Study flowchart describing the details of the randomized controlled trial. The entire trial includes assessments on week 1, week 4, and week 8 (follow-up visit).

This study was designed as an investigator-initiated, double-blind, randomized, placebo-controlled clinical trial to assess the efficacy and safety of SCRT in patients with AD and respiratory symptoms. A total of 60 participants will be divided into an SCRT group and a placebo group at a 1:1 ratio and the study drugs will be administered for 4 weeks. At weeks 4 and 8 of follow-up, the participants will visit the study institute and be assessed the efficacy and safety (including the onset of adverse events). Specific schedules and investigations are summarized in Table 1 and Figure 1. The protocol (version 1.1) follows standard protocol items: recommendation for interventional trials guideline.

**Table 1:**
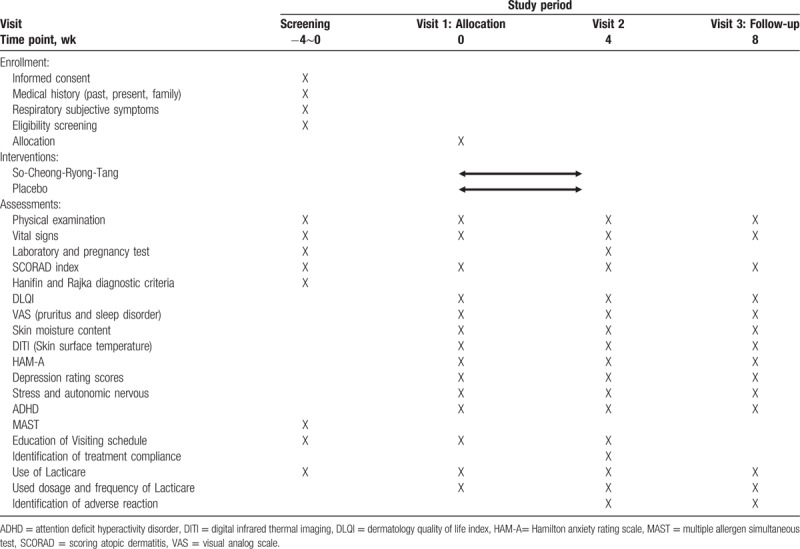
Schedule of enrolment, interventions, and assessments.

### 2.2 Participants

#### 2.2.1 Recruitment

The participants are expected to be recruited through the Wonkwang University Oriental Medical Hospital at Iksan from June 2019 to August 2020. Advertisement for participating in the study will be put up on the bulletin boards of the hospital, city, shopping complexes, and apartments. During the screening, the investigators will explain all the necessary information related to the trial and predictable outcomes to the participants and their guardians and obtain their signatures on the consent form. The participants can withdraw from the study at any time without any problem. The written consent contains the study background, objective, investigational drugs and placebo, expected results, and predicted benefits and harms.

#### 2.2.2 Inclusion criteria

The subjects of this study should meet all the following criteria:

(1) Male and female patients between 7 and 65 years of age, scoring 15 to 50 in a scoring atopic dermatitis (SCORAD), and having more than 3 of major criteria and 3 of minor criteria based on the Hanifin and Rajka diagnostic criteria
(2) Patients marking more than 1 respiratory disease and symptoms in the questionnaire survey for respiratory disorders
(3) Those who fully understand our study after detailed explanation, and voluntarily agree to participate and provide written consent to undertake precautions (in case of a child, both the child and the parent should sign the consent form)

#### 2.2.3 Exclusion criteria

(1) Patients undergoing intensive drug treatment for severe AD (antihistamine, adrenocortical hormone, herbal drugs, etc)
(2) Patients using an oral antihistamine, steroid, antibiotic, systemic photochemotherapy, or another immunosuppressant within 4 weeks before initiation of the trial
(3) Patients with systemic infection or undergoing systemic antibiotic treatment
(4) Patients with a skin disease other than AD, pigmentation, or a large scar around the AD site
(5) Patients taking interferon drugs (interferon-alpha, interferon-beta, etc)
(6) Patients with liver diseases, such as cirrhosis and liver cancer
(7) Patients with a platelet count of <100,000/mm^3^ due to liver dysfunction caused by chronic hepatitis
(8) Patients with kidney diseases, such as acute/chronic renal failure, and nephrotic syndrome
(9) Patients with acute severe cardiovascular diseases, such as heart failure, myocardial infarction, and stroke
(10) Patients with chronic diseases, such as uncontrolled hypertension, diabetes, gastrointestinal disorder, hyperhidrosis, and dysuria
(11) Patients taking potassium-containing drugs, licorice-containing drugs, glycyrrhizinate or its salt-containing drugs, loop diuretics (furosemide, ethacrynic acid), or thiazide diuretics (trichloromethiazide) within 4 weeks before initiation of the trial
(12) Patients taking ephedra or ephedrine-containing drugs, monoamine oxidase inhibitors, drugs for thyroid disorders, catecholamines, or xanthine drugs within 4 weeks before initiation of the trial
(13) Patients taking antipsychotic drugs within 2 months before screening
(14) Patients who participated in any other clinical trial within the preceding 4 weeks
(15) Patients with previous hypersensitivity reaction to our investigational drug or its components
(16) Patients with a history of drug or alcohol abuse
(17) Women who are pregnant or lactating
(18) Among fertile women with a chance of being pregnant, those who do not practice proper contraception (excluding women who underwent surgical sterilization)
(19) Patients with thyroid disorders with thyroid-stimulating hormone (TSH) level is ≤0.35 μU/mL or ≥4.94 μU/mL at screening
(20) Patients determined to be unsuitable for participating in this study by the principal investigator due to the results of the diagnostic tests or any other reasons.

### 2.3 Study phase

During screening at the hospital, the eligibility of the participants will be adjudicated based on their medical history, medication history, results of blood and urine tests, vital signs, SCORAD index, and Hanifin and Rajka diagnostic criteria. Then the participants determined to be eligible for the trial will visit the hospital (visit 1) within 2 weeks after screening and will be randomly assigned to the treatment or the control group. After group allocation, various surveys including AD survey and physical examination will be conducted, and the participants will be administered with the investigational drug 3 times a day for 4 weeks. After 4 weeks, the participants will be asked to visit the hospital again (visit 2) and undertake the same surveys, examinations, and blood investigations as those at visit 1. For the next 4 weeks, all drugs will be withdrawn except Lacticare, a concomitant drug, and the participants will be asked to visit the hospital (visit 3) and undertake the same surveys and investigations. The specific schedules and types of examinations are summarized in Table 1.

### 2.4 Intervention

Experimental group and control group will be orally administered 1 packet of either SCRT or placebo 3 times a day before meals for 4 weeks. Based on the adult dosage of commercial SCRT extract granule (3 g thrice daily), the dose was set as 9.0 g/d for patients of 15 to 65 years of age. The dose in patients between 7 and 14 years of age was set as 6.0 g/d (two-third of the adult dosage) based on the Oriental and Herbal Medicine Pharmaceutical Approval Article 16 Usage and Dose No. 7. SCRT and placebo are brown granules of the same color, dosage form, and physical property. The ingredients of each drug are shown in the Table 2. The manufacturer of the drugs is Hanpoong Pharm (Hyoryeong-ro, Seocho-gu, Seoul, Republic of Korea). The drugs will be stored in an airtight container at room temperature (1–30°C). The concomitant medication for the experimental and control groups is hydrocortisone acetate 1%/Lacticare ointment (100 g of the ointment contains 1.0 g of the main ingredient hydrocortisone, 0.15 g of methyl ρ-hydroxybenzoate, and 0.05 g of propyl ρ-hydroxybenzoate) which can be used during the trial period of 8 weeks. The participants will record the number of drug administration and the corresponding dosage.

**Table 2:**
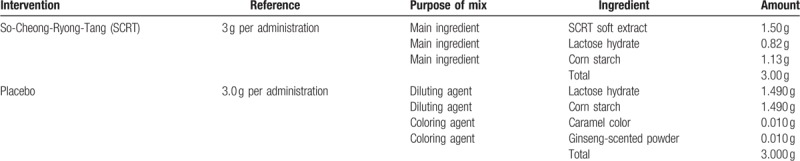
Composition of So-Cheong-Ryong-Tang and placebo.

#### 2.4.1 Concomitant medications

(1) Product (code) name and manufacturer: Lacticare HC lotion 1% (hydrocortisone), Korean Pharma Inc,
(2) Dosage form and property: white lotion.

### 2.5 Randomization

The participants who are willing to provide written consent to participate in this trial will be given a screening number according to the order of their written consent. The participants determined to be eligible for the trial following the inclusion and exclusion criteria will be randomly assigned a number in the order of their visit dates, and the treatment for each participant will be prescribed according to the random code generated. For randomization, block randomization will be used with block sizes of 4, 6, or 8 with a set ratio of 1:1. After generating the randomization table, drugs for each patient will be packaged according to the table, labeled according to the randomization code, and transported to the pharmacist manager or herb pharmacist in the clinical trial research institute. Then, the pharmacist manager or herb pharmacist will dispense the drugs after matching the randomization codes to participants.

### 2.6 Blinding

To obtain scientific results from the clinical trial and to avoid bias, the investigators and participants will be double-blinded so that they will be unable to know the groups to which the individual participants are assigned. The double-blinding will be maintained unless any medical emergency occurs. The information regarding the assigned groups will be locked and will be divulged only during statistical analysis after the whole clinical trial process is completed. When the allocation code will be broken due to adverse events, it will be reported to the institutional review board and the client following the procedures of code-breaking.

### 2.7 Sample size calculation

The primary objective of this investigator-initiated clinical trial is to demonstrate a larger reduction in the AD symptom in the experimental group than the placebo group after 4 weeks of treatment and to validate the efficacy of SCRT in AD-related respiratory symptoms. To calculate the sample size, we anticipated that the mean changes in SCORAD scores after 4 weeks was 13.8 (*μt*) in the SCRT group and 7.9 (*μc*) in the placebo group. The standard deviation (*σ*) between the SCRT and placebo groups was assumed to be 8.2. The type 2 error at a 5% significance level was set as 0.2 and the power of the test was maintained at 80%. Taking a 20% dropout rate into account and maintaining the 1:1 allocation ratio between the SCRT and placebo groups, the final sample size was 30 in each group.

### 2.8 Outcome measures and schedule

#### 2.8.1 Evaluation of the primary outcome

The primary outcome of the clinical trial is the changes in SCORAD total scores and sub-scores at week 4 as compared to that at the baseline.

#### 2.8.2 Evaluation of the secondary outcomes

The secondary outcomes of the clinical trial are the changes in the dose and frequency of usage of the AD ointment, dermatology life quality index scores, pruritus and sleep disorder in visual analog scale, skin moisture content, skin surface temperature, Hamilton anxiety rating scale scores, depression rating scale scores, stress/autonomic nervous function test, and attention deficit hyperactivity disorder survey scores at week 4 as compared to those at the baseline.

### 2.9 Statistical analyses

Appropriate statistical tests will be performed according to the characteristic of the data, the number of associated variables and their correlations, and the state of distribution. Windows Strategic applications software will be used for analysis. At a significance level of 5% and power of 80%, 95% confidence interval will be reported for the differences in the results between the groups. Participants with results of at least 1 efficacy outcome after administration of the investigational drug after randomization will be included for efficacy assessment. In addition, all subjects who will receive the investigational drug at least once and visit the hospital will be included for the safety assessment. Chi-square test or Fisher exact test will be used for the discrete variables, and analysis of variance or Kruskal–Wallis test will be used for the continuous variables.

### 2.10 Efficacy assessment

Changes in the assessment items at week 4 as compared to the baseline in each group will be presented, and the differences between the experimental and control groups will be analyzed using the independent *t* test. Additionally, changes in the assessment items at week 4 as compared to before drug administration in each group will be analyzed using the paired *t* test. The baseline variables for each efficacy outcomes and non-homogenous demographic variables will be adjusted according to the covariates and will be used for analysis of covariance.

### 2.11 Safety assessment

(1) Adverse events: All adverse events reported throughout the trial period will be noted and the prevalence rate of the adverse events will be calculated. The ratio of the participants showing adverse events between the groups will be calculated and analyzed using the Chi-square test or Fisher exact test.
(2) Laboratory test results: The means and standard deviations of the laboratory test results will be presented and the changes from the baseline will be compared using paired *t* test or Wilcoxon signed-rank test.
(3) Vital signs: The means and standard deviations of blood pressure and heart rates measured at each visit will be presented and the patterns of significant changes in each item from the initial treatment to completion will be compared using paired *t* test or Wilcoxon signed-rank test.

### 2.12 Data management

The investigators will record all data collected during this clinical trial in the case report form provided by the client, and the clinical trial manager will keep a copy of the case report form. When there are missing data, the investigators will leave a remark with proper explanation. While making changes to the case report form, the original record will be recognizable and the clinical trial manager making such changes should sign it with the date. All the above information will be stored and managed by the AD Research Center at Wonkwang University Korean Medical Hospital, Iksan. In observation of the Personal Information Protection Act, the names of the participants will be kept confidential and the participants will be denoted only by the numbers assigned. The participants will be informed that all the clinical trial data will be stored in a computer and will be kept strictly confidential. The signed written consent will be stored by the clinical trial manager.

### 2.13 Monitoring

For the trial to be adherent to the Korea Good Clinical Practice and for the trial data to be acknowledged upon registration both domestically and internationally, a Contract Research Organization, a specialized institute for monitoring, will monitor the entire process of the trial to assure accuracy and reliability of the data and improve the quality of clinical research. To confirm completeness and clarity of the case record forms, the source document will be compared and reviewed with the case record forms in the presence of the investigators, and they should always cooperate with the client. The data recorded in the protocol that are basic items required by International Conference on Harmonization's Good Clinical Practices (ICH-GCP) will be consistent with the source document and should be reported accurately. Any dose and/or therapy modifications, adverse events, history of concomitant medications, intercurrent illnesses developed during the trial period, and reasons for withdrawals and dropouts will be monitored. The monitoring visit reports will be composed of a summary of the visit, narrative description, issues, and checklist results.

### 2.14 Safety and adverse events

If the participants experience adverse events during the trial, the investigators will record verified items, such as the date of onset, date of recovery, association with the investigational drugs, related measures and treatment, and results in the case report form according to the name and severity of the adverse events. Until recovery or stabilization from the adverse events or verification that the adverse events were caused by the investigational drugs or factors unrelated to the clinical trial, follow-up on the adverse events will be continued. Abnormal laboratory test results and abnormal vital signs that are determined to be clinically significant will be recorded as adverse events. For safety assessment, blood and urine tests will be conducted during the screening visit and visit 2 after administration of the investigational drugs, and any physically abnormal signs will be observed before and after drug administration. Furthermore, through visit 3 (1 month after the completion of drug administration), follow-up will be continued to detect any adverse events.

### 2.15 Ethical aspects

This trial will be conducted according to the Declaration of Helsinki, ICH-GCP, and all other applicable domestic and international regulations. It has been approved by the institutional review board of the Wonkwang University Korean Medicine Hospital, Iksan. SCRT, the investigational drug of our study, was approved for treating AD by the Director of Korea's Ministry of Food and Drug Safety.

## 3 Discussion

This randomized, double-blinded, placebo-controlled trial protocol is designed to assess the efficacy and safety of SCRT on AD patients with respiratory disorders. We will evaluate the efficacy and safety of SCRT as compared to placebo after 4 weeks of treatment, and we will recruit patients aged between 7 and 65 years and having AD and respiratory disorders. In our previous study, we categorized etiology of the AD into those with gastrointestinal factor and respiratory factor.^[19]^ Accordingly, Soshiho-Tang was administered to the patients with AD and gastrointestinal disorders in our previous clinical trial.^[20]^ As a follow-up study, we designed this randomized, double-blinded, placebo-controlled trial to assess the efficacy and safety of SCRT in AD patients with respiratory disorders.

Atopic march is the concept that describes the progression of AD to allergic rhinitis and asthma.^[21]^ Meanwhile, it has been suggested that a dysfunctional immune system can results in eczema, food allergy, and airway allergies so that it is not only a concept of progression but also diverse reactions to cutaneous sensitization through an impaired barrier in the skin, gut, and lungs.^[22]^ Thus, the pathological basis of different diseases can be the same and the therapeutic approach can be taken from the same point of view. Based on this perspective, we expect that SCRT will be effective and safe for the treatment of AD and respiratory disorders considering that SCRT has been already used for the treatment of allergic rhinitis and bronchial asthma in the past.

Although AD is generally known to have a high prevalence in children, the persistence of AD in adulthood is also common and it affects the quality of life.^[23]^ For this reason, adults of <65 years of age are also included in this study, unlike the previous study which included only the children and adolescents. We hope that the results of this trial will provide evidence for the use of SCRT as a new treatment for AD with respiratory disorders.

## Author contributions

**Conceptualization:** Su-Jin Kang.

**Data curation:** Su-Jin Kang.

**Funding acquisition:** Min-Cheol Park.

**Methodology:** Eun-Heui Jo.

**Project administration:** Min-Cheol Park.

**Supervision:** Han-Baek Cho.

**Validation:** Min-Cheol Park.

**Visualization:** Geum-Jin Yang, Ji-Eun Hong.

**Writing – original draft:** Su-Jin Kang.

**Writing – review and editing:** Han-Baek Cho, Eun-Heui Jo, Geum-Jin Yang, Ji-Eun Hong, Ju-Hyun Lee, Yu-Hwa Shim, Jung-Hyun Mun, Min-Cheol Park.

## References

[1] Wollenberg A Barbarot S Bieber T Consensus-based European guidelines for treatment of atopic eczema (atopic dermatitis) in adults and children: part I. Eur Acad Dermatology Venereol 2018;32:657–82. 10.1111/jdv.14891 29676534

[2] Nutten S Atopic dermatitis: global epidemiology and risk factors. Ann Nutr Metab 2015;66:8–16. 25925336 10.1159/000370220

[3] Kiister W Petersen M Christophers E A family study of atopic dermatitis clinical and genetic characteristics of 188 patients and 2151 family members. Arch Dermatol Res 1990;282:98–102. 2353830 10.1007/BF00493466

[4] Bantz SKB Zhu Z Zheng T The atopic march: progression from atopic dermatitis to allergic rhinitis and asthma. J Clin Cell Immunol 2014;5:1–6. 10.4172/2155-9899.1000202 PMC4240310 25419479

[5] Otsuka A Nomura T Rerknimitr P The interplay between genetic and environmental factors in the pathogenesis of atopic dermatitis. Immunol Rev 2017;278:246–62. 28658541 10.1111/imr.12545

[6] Leung DYM Pathogenesis of atopic dermatitis. J Allergy Clin Immunol 1999;104:99–108. 10.1016/s0091-6749(99)70051-5 10482860

[7] William L Weston MdwH Treatment of atopic dermatitis (eczema). UpToDate. Published 2019. Available at: https://www.uptodate.com/contents/treatment-of-atopic-dermatitis-eczema. Accessed October 8, 2019.

[8] Yu S Drucker AM Lebwohl M A systematic review of safety and efficacy of systemic corticosteroids in atopic dermatitis. J Am Dermatol 2018;78:733–40. 10.1016/j.jaad.2017.09.074 29032119

[9] Yuan H Ma Q Ye L The traditional medicine and modern medicine from natural products. Molecules 2016;21:559. 10.3390/molecules21050559 PMC6273146 27136524

[10] Chin H-W Jang H-S Jang B-S A study on utilization of alternative medicine for patients with atopic dermatitis. Korean Dermatological Assoc 2005;43:903–11.

[11] Jung SC Sun-Jin M Kyoung-II K Effects of Socheongryong-Tang on immunoglobulin production in asthmatic mice. Korea Assoc Herbol 2008;23:23–8.

[12] Shimada T Kondoh M Motonaga C Enhancement of anti-allergic effects mediated by the Kampo Medicine Shoseiryuto (Xiao-Qing-Long-Tang in Chinese) with lysed enterococcus faecalis FK-23 in Mice. Asian Pacific J Allergy Immunol 2010;28:59–66. 20527518

[13] Sakaguchi M Mase A Iizuka A Further pharmacological study on Sho-seiryu-to as an antiallergic. Methods Find Exp Clin Pharmacol 1997;19:707–13. 9542721

[14] Tanno Y Shindoh YTT Modulation of human basophil growth in vitro by xiao-qing-long-tang (syo-seiryu-to), chai-pu-tang (saiboku-to), qing-fei-tang (seihai-to), baicalein and ketotifen. Am J Chin Med 1989;17:45–50. 2573996 10.1142/S0192415X89000085

[15] Yamahara J Yamada T Kimura H Biologically active principles of crude drugs. Anti-allergic principles of “Shoseiryu-to.” I. Effect on delayed-type allergy reaction. Yakugaku Zasshi 1982;102:881–6. 7153865 10.1248/yakushi1947.102.9_881

[16] Ko E Rho S Lee E Traditional Korean medicine (SCRT) modulate Th1/Th2 specific cytokine production in mice CD4 + T cell. J Ethnopharmacol 2004;92:121–8. 15099858 10.1016/j.jep.2004.02.008

[17] Murata K Toriumi Y Kamei T Effect of Sho-Seiryu-To (XIAO-QING-LONG-TANG) on skin itching and peripheral eosinophil level in three elderly patients. Orient Pharm Exp Med 2005;5:167–71.

[18] Hiroyuki M Hisao S Harukiyo O Primary skin care part (III) control of atopic dermatitis and skin itching by combined remedies of Shoseiryuto, Tsumura 19 and repeated electrical stimulation through the acupuncture needle at electrodermal points (=Nakatani's Ryodtoen). Japanese Soc Ryodouraku Med 1982;27:209–13.

[19] Park S Noh H Hwang C Classification of atopic dermatitis into digestive and respiratory disorders on the basis of a literature study. J Korean Med Ophthalmol Otolaryngol Dermatol 2016;29:106–23.

[20] Kang S Jo E Yang G Efficacy and safety of Soshiho-tang in patients with atopic dermatitis and gastrointestinal disorders. Study protocol for a double-blind, randomized, and placebo-controlled clinical trial. Medicine (Baltimore) 2019;98:e15479. 31045830 10.1097/MD.0000000000015479 PMC6504306

[21] Spergel JM Paller AS Atopic dermatitis and the atopic march. J Allergy Clin Immunol 2003;112:S118–27. 14657842 10.1016/j.jaci.2003.09.033

[22] Johansson E Hershey GKK Contribution of an impaired epithelial barrier to the atopic march. Ann Allergy Asthma Immunol 2019;120:118–9. 10.1016/j.anai.2017.11.008 PMC6033259 29413333

[23] Mortz CG Andersen KE Dellgren C Atopic dermatitis from adolescence to adulthood in the TOACS cohort: prevalence, persistence and comorbidities. Allergy Eur J Allergy Clin Immunol 2015;70:836–45. 10.1111/all.12619 25832131

